# Snakes on an African plain: the radiation of *Crotaphopeltis* and *Philothamnus* into open habitat (Serpentes: Colubridae)

**DOI:** 10.7717/peerj.11728

**Published:** 2021-08-06

**Authors:** Hanlie M. Engelbrecht, William R. Branch, Krystal A. Tolley

**Affiliations:** 1 School of Animal, Plant and Environmental Sciences, University of the Witwatersrand, Johannesburg, Gauteng, South Africa; 2 Kirstenbosch Research Centre, South African National Biodiversity Institute, Cape Town, Western Cape, South Africa; 3 Department of Botany & Zoology, Stellenbosch University, Cape Town, Western Cape, South Africa; 4 Herpetology, Port Elizabeth Museum (Bayworld), Port Elizabeth, Eastern Cape, South Africa; 5 Department of Zoology, Nelson Mandela Metropolitan University, Port Elizabeth, Eastern Cape, South Africa

**Keywords:** Sub-Saharan Africa, Forest contraction, Savanna expansion, Green and Bush Snakes, Herald Snakes, Lineage diversification, Ancestral habitat state reconstruction

## Abstract

**Background:**

The African continent is comprised of several different biomes, although savanna is the most prevalent. The current heterogeneous landscape was formed through long-term vegetation shifts as a result of the global cooling trend since the Oligocene epoch. The overwhelming trend was a shift from primarily forest, to primarily savanna. As such, faunal groups that emerged during the Paleogene/Neogene period and have species distributed in both forest and savanna habitat should show a genetic signature of the possible evolutionary impact of these biome developments. *Crotaphopeltis* and *Philothamnus* (Colubridae) are excellent taxa to investigate the evolutionary impact of these biome developments on widespread African colubrid snakes, and whether timing and patterns of radiation are synchronous with biome reorganisation.

**Methods:**

A phylogenetic framework was used to investigate timing of lineage diversification. Phylogenetic analysis included both genera as well as other Colubridae to construct a temporal framework in order to estimate radiation times for *Crotaphopeltis* and *Philothamnus*. Lineage diversification was estimated in Bayesian Evolutionary Analysis Sampling Trees (BEAST), using two mitochondrial markers (cyt–*b*, ND4), one nuclear marker (c–mos), and incorporating one fossil and two biogeographical calibration points. Vegetation layers were used to classify and confirm species association with broad biome types (‘closed’ = forest, ‘open’ = savanna/other), and the ancestral habitat state for each genus was estimated.

**Results:**

*Philothamnus* showed an ancestral state of closed habitat, but the ancestral habitat type for *Crotaphopeltis* was equivocal. Both genera showed similar timing of lineage diversification diverging from their sister genera during the Oligocene/Miocene transition (*ca*. 25 Mya), with subsequent species radiation in the Mid-Miocene. *Philothamnus* appeared to have undergone allopatric speciation during Mid-Miocene forest fragmentation. Habitat generalist and open habitat specialist species emerged as savanna became more prevalent, while at least two forest associated lineages within *Crotaphopeltis* moved into Afromontane forest habitat secondarily and independently.

**Discussion:**

With similar diversification times, but contrasting ancestral habitat reconstructions, we show that these genera have responded very differently to the same broad biome shifts. Differences in biogeographical patterns for the two African colubrid genera is likely an effect of distinct life-history traits, such as the arboreous habits of *Philothamnus* compared to the terrestrial lifestyle of *Crotaphopeltis*.

## Introduction

Epochal climatic oscillations were one of the most influential abiotic factors that contributed to progressive change of the African landscape. Habitat transformations included spatial shifts of major biomes and the emergence of novel habitats (e.g., Axelrod & Raven, 1978; Hewitt, 2004a). For example, the landscape of the African continent has transformed from a nearly pan-African forest since the Eocene (Bobe, 2006; Couvreur et al., 2008; Couvreur et al., 2021; Kissling et al., 2012) to primarily savanna at present (Edwards et al., 2010). During the Early and Middle Miocene a heterogeneous landscape developed, incorporating both open (e.g., savanna) and closed (i.e., forest) habitats as a result of the global decline in temperature and greater rainfall seasonality (Shipman et al., 1981; Retallack, Dugas & Bestland, 1990; Cerling et al., 1991; Zachos et al., 2001; Bobe, 2006; Couvreur et al., 2008; Couvreur et al., 2021; Kissling et al., 2012). These conditions favoured grass-dominated ecosystems (e.g., savanna) in many regions of the continent during the Pliocene-Pleistocene, with wet and dry alternation of African climate, punctuated by periods of increased aridity (Cerling, 1992; Bobe & Behrensmeyer, 2004; Wynn, 2004; Bobe, 2006; Kissling et al., 2012). The progression throughout the Cenozoic therefore, has been from a continent dominated by closed forest habitats, toward open habitat (e.g., savanna). Additional factors that influenced the formation of open habitat were the interaction between geological processes, edaphic and phytogeographical dynamics, and factors such as herbivory, fire, and in modern times, human cultural practices (Huntley, 1982). Topography, affected moisture transport such as rainfall patterns, as well as hydrological modifications that could have induced strong shifts in vegetation (Sepulchre et al., 2006).

The impact of the biome reorganisation on the evolution of African faunal groups is relatively well documented for mammals and birds (e.g., Pomeroy & Ssekabiira, 1990; Fjeldsa & Lovett, 1997; Barnett et al., 2006; Brown et al., 2007; Dehghani et al., 2008; Ishida et al., 2011; Miller et al., 2011; Lorenzen, Heller & Siegismund, 2012; Levinsky et al., 2013; Smitz et al., 2013). A notable trend of increased diversity of large herbivorous mammals is linked to the growing dominance of open habitat formation during the Late Miocene to Early Pleistocene (Kappelman et al., 1997; Bobe & Behrensmeyer, 2004; Bobe, 2006; Edwards et al., 2010). Similarly, palaeo-environment induced lineage diversification has been demonstrated for some African reptile taxa for example, African cobras (Wüster et al., 2007), vipers (Barlow et al., 2013; Menegon et al., 2014; Barlow et al., 2019), semi-aquatic varanids (Dowell et al., 2016), skinks (Medina et al., 2016) and chameleons (Tolley, Chase & Forest, 2008; Tolley et al., 2011; Ceccarelli et al., 2014). These studies show that since the Oligocene, some African reptile groups have undergone lineage diversification, particularly throughout the Miocene period. Such common patterns for diverse reptile taxa suggest that there could be some general processes (for example the concomitant contraction and expansion of the forest and savanna biomes during the Miocene period) that played a key role in African reptile evolution.

African reptile genera that emerged prior to the Miocene period and have species distributed in both forest and savanna habitat should show a genetic signature of the possible evolutionary impact of these biome developments. The snake family, Colubridae, appears to have diverged from their sister clade, (Elapidae + Lamprophiidae), during the Eocene suggesting that genera within Colubridae are younger than 50 Mya (Zheng & Wiens, 2016). The African colubrid snake genera, *Crotaphopeltis* and *Philothamnus* are widespread throughout sub-Saharan Africa and their species are mainly associated with either forest or savanna. They are therefore good candidates to test drivers of speciation with respect to major biome reorganisation. Accordingly, divergence of *Crotaphopeltis* and *Philothamnus* from their forest-living sister groups (*Dipsadoboa* and *Hapsidophrys*, respectively) and radiation into open habitat could have been initiated during the Oligocene/Miocene transition, when habitats became more heterogeneous. Alternatively, speciation could have been influenced by the dramatic contraction of forest in the Mid-Miocene (Kissling et al., 2012). Species in both genera possibly radiated from closed habitat to open habitat, as the landscape transformed from predominant forest habitats to primarily savanna habitat. Therefore, multiple shifts of species from closed to open habitat probably occurred.

Here, the temporal and spatial evolutionary patterns of *Crotaphopeltis* and *Philothamnus* were examined. Synchronised timing of lineage diversification in response to habitat contraction and expansion events, as well as radiation patterns of species into open habitat were investigated and compared between the two genera. In particular, we hypothesised that 1) *Crotaphopeltis* and *Philothamnus* diverged from their forest relatives, followed by speciation and the emergence of habitat generalist species in both genera during the Oligocene/Miocene transition (*ca*. 24 Mya) when habitats became more heterogeneous. Furthermore, 2) savanna specialists emerged since the Mid-Miocene (*ca*. 16–11 Mya) when open habitats became widespread. Therefore, multiple shifts from closed to open habitat, for species within both genera are expected since the Late Oligocene. Timing of lineage diversification estimation and ancestral state reconstruction methods were used to investigate the biogeographic history of *Crotaphopeltis* and *Philothamnus* with respect to the evolution of the African forest and savanna biomes.

## Material and Methods

### Background

The species and putative species identified within *Philothamnus* (Engelbrecht et al., 2019) and *Crotaphopeltis* (Engelbrecht et al., 2020) were used to estimate diversification dates and to reconstruct ancestral habitats. Our analyses therefore include the currently recognised species as well as undescribed species (i.e., lineages that show species level divergence but are not yet described as species). We refer to both the described and undescribed species as ‘taxa’ throughout. Outstanding taxonomic issues are that *Philothamnus semivariegatus* is paraphyletic, comprising four cryptic lineages, *P. carinatus* contains two cryptic lineages while *P. dorsalis* and *P. giraradi* appear to be a single taxon that require synonymising (Engelbrecht et al., 2019). Similarly, previous analyses of *Crotaphopeltis* indicated that *Crotaphopeltis tornieri* is not monophyletic, and consists of at least two separate taxa that are not sister clades (Engelbrecht et al., 2020).

*Philothamnus* are arboreal snakes, and the genus is widely distributed across the Afrotropical realm (Table S1). The taxa show variation with regards to the level of habitat specialisation and can be divided into habitat classes in which they typically occur. *Philothamnus* taxa were coded according to their primary habitat –forest species: *P*. *carinatus* 1, *P*. *carinatus* 2, *P. heterodermus*, *P. natalensis*, *P*. *nitidus*, *P*. *ruandae*, *P*. *thomensis*, savanna species: *Philothamnus ornatus*, *P. semivariegatus* 1, generalists (both habitat types): *P*. *angolensis*, *P*. *dorsalis/girardi*, *P*. *hoplogaster*, *P*. *macrops*, *P*. *occidentalis*, *P*. *punctatus*, *P. semivariegatus* 2, *P. semivariegatus* 3, *P. semivariegatus* 4 (Broadley, 1983; Loveridge, 1958; Hughes, 1985; Trape & Baldé, 2014; Wallach, Williams & Boundy, 2014). Samples of *P*. *battersbyi*, *P*. *belli*, *P*. *bequaerti*, *P*. *heterolepidotus*, *P*. *hughesi*, *P*. *irregularis*, *P*. *pobeguini*, and *P*. *s*. *smithi* were not available for inclusion in the analyses.

*Crotaphopeltis* are terrestrial snakes which also show variation with regards to the level of habitat specialisation and can be divided into habitat classes in which they typically occur: *C. barotseensis* occupies moist grasslands, *C. tornieri* 1 and *C. tornieri* 2 in Afromontane forest, *C. degeni* in grassland, savanna and shrublands, and the widespread *C. hotamboeia*, occurs in all habitats except tropical rainforest and arid regions (Table S1; Broadley, 1983; Rasmussen, 1993; Rasmussen & Hughes, 1997; Rasmussen, Chirio & Ineich, 2000; Wallach, Williams & Boundy, 2014). *Crotaphopeltis braestrupi* and *C*. *hippocrepis* were not available for inclusion.

### Lineage divergence dating

The subfamily Colubrinae is paraphyletic with *Crotaphopeltis* and *Philothamnus* forming part of the subfamily “Colubrinae 2” *sensu* Zheng & Wiens (2016). Therefore, to estimate timing of lineage diversification within the two genera, a phylogeny was constructed including *Crotaphopeltis* and *Philothamnus* plus additional colubrid genera from “Colubrinae 2”. Species and genera were chosen so that fossil calibration points could be used in the analysis (Table S2), including sister genera (*Dipsadoboa unicolor* and *Hapsidophrys* spp*.* for *Crotaphopeltis* and *Philothamnus*, respectively). *Crotaphopeltis* and *Philothamnus* were represented by taxa within the respective genera (Engelbrecht et al., 2019; Engelbrecht et al., 2020). Representative species from closely related Colubridae subfamilies (Grayiinae, Calamariinae and Sibynophiinae) were used as outgroup taxa (Pyron et al., 2014; Zheng & Wiens, 2016).

Diversification dates within *Crotaphopeltis* and *Philothamnus* were estimated in Bayesian Evolutionary Analysis Sampling Trees (BEAST, v. 2.3.0, Bouckaert et al., 2014), using data for three genetic markers previously generated by Engelbrecht et al. (2019) and Engelbrecht et al. (2020). The total evidence dataset comprised 555 bp for cytochrome *b* (cyt–b), 650 bp for nicotinamide adenine dehydrogenase subunit four (ND4) and 514 bp for oocyte maturation factor Mos (c–mos), for 5 *Crotaphopeltis* (*n* = 16) and 18 *Philothamnus* (*n* = 29) taxa (Table S1). Sequences for 37 additional colubrid taxa were retrieved from GenBank (Table S2). The Bayesian framework uses a probabilistic model to define rates of molecular sequence evolution over time, on an unconstrained phylogeny, using the MCMC methods to derive clade ages. Likelihood ratio tests, implemented in MEGA-X (Kumar et al., 2018), rejected the strict molecular clock for all data partitions. Therefore, an uncorrelated lognormal molecular clock and the reversible-jump based substitution model of Bouckaert, Alvarado-Mora & Pinho (2013) were employed. For each gene partition, a gamma distribution of among-site rate heterogeneity with four rate categories was assumed. A Yule speciation process was imposed. A total of three calibration points were available (Nagy et al., 2003; Wüster et al., 2007; Pook et al., 2009; Tamar et al., 2016; Head, Mahlow & Müller, 2016): (C1) *Hemorrhois* divergence between the eastern (*H. ravergieri* and *H. nummifer*) and western (*H. algirus* and *H. hippocrepis*) subclades that occurred after the contact of Africa-Arabia with Eurasia, 16–18 Mya (Nagy et al., 2003), (C2) *Hierophis* subclade divergence, including *Eirenis* 18 Mya according fossil data (Ivanov, 2002), and (C3) a *Pantherophis* fossil with a minimum age of 11.93 Mya, calibrating the divergence between the extant genera *Pantherophis* and *Pituophis* (Head, Mahlow & Müller, 2016). A normal distribution prior with a mean of 18 Mya and standard deviation of 2 Mya was applied to the biogeographical calibration, C1 (95% confidence interval of 14.7–21.3 Mya), as well as a normal distribution prior for C2 with a mean of 18 Mya and standard deviation of 1 Mya (95% confidence interval of 16.4–19.6 Mya). A lognormal prior was applied to C3, using the fossil age, 11.93 Mya as zero-offset, the default lognormal mean of 1 and standard deviation of 1, to specify a mode slightly older than the fossil in order to model the likelihood of actual date of the node (Ho & Phillips, 2009). Ten independent runs of the Monte Carlo Markov-chain (MCMC) were made, each for 100 million generations, sampled every 100,000 generations. Convergence of the ten independent runs was verified by the effective samples size for each parameter in TRACER v. 1.6.0 (Rambaut & Drummond, 2007) after a 10% burn-in. The post-burn in set of trees from the ten runs were combined using LOGCOMBINER v. 2.3.0 (Rambaut & Drummond, 2014a) and TREEANNOTATER v. 1.8 (Rambaut & Drummond, 2014b) with the final chronogram and node ages visualised in FigTree v. 1.3.1 (Rambaut, 2010).

### Ancestral habitat state reconstruction

**Figure 1:**
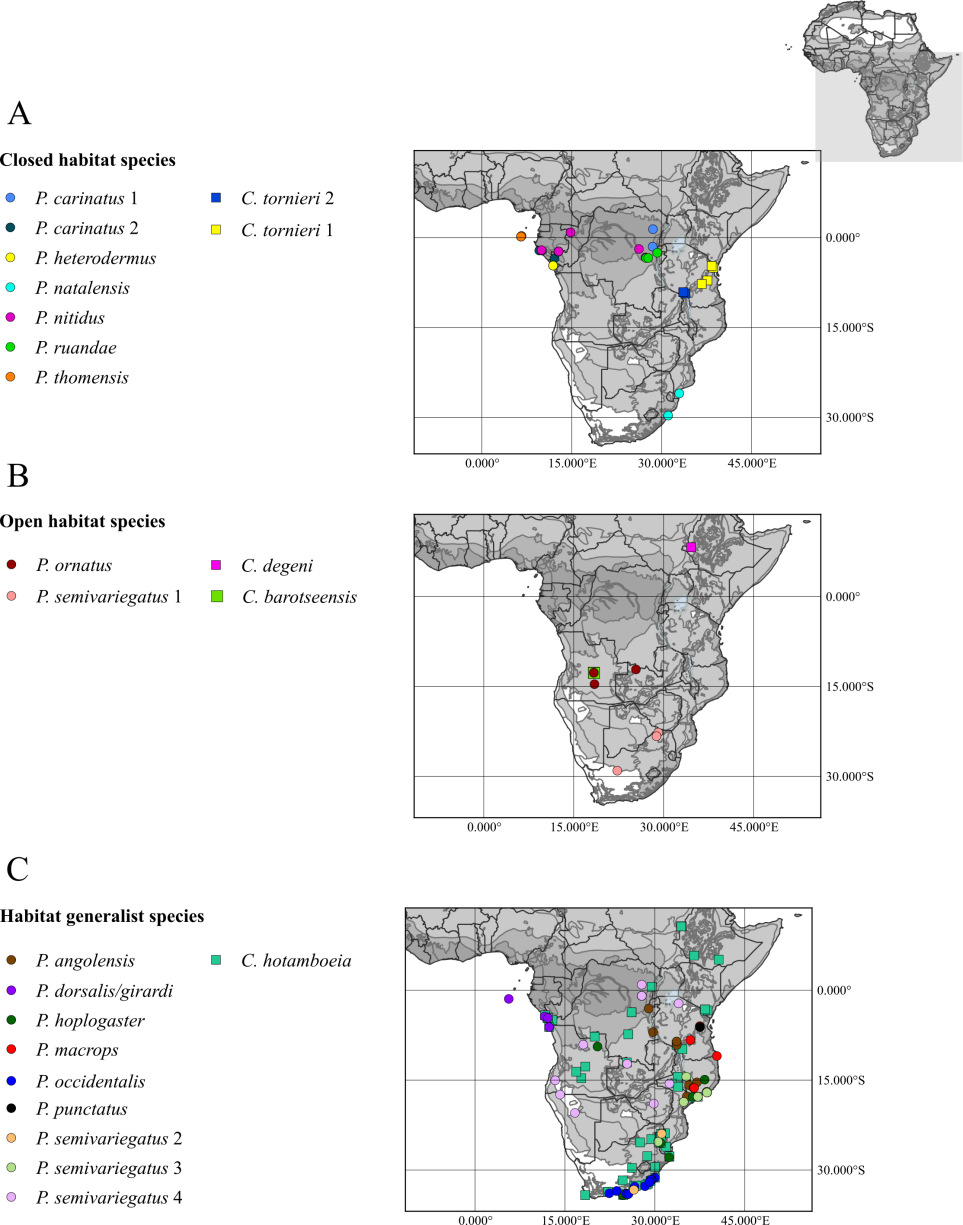
Habitat classification for genetic samples of *Crotaphopeltis* and *Philothamnus* species. Sampled localities were projected onto the biome map of Olson et al. (2001). Closed habitat (forest classes) is indicated by darker grey shades. Open habitat (including savanna) is shaded white to light grey.

Ancestral habitats were reconstructed for *Crotaphopeltis* and *Philothamnus* separately, using likelihood optimisation and the Markov *k-* state, one-parameter model (all states equally probable) in MESQUITE v. 3.03 (Maddison & Maddison, 2015). The ultrametric BEAST tree was pruned for *Crotaphopeltis* and *Philothamnus* separately and species from closely related sister genera for each genus were retained to allow for habitat state at the base of the clade to be estimated in the ancestral habitat reconstruction analysis. Biome classifications were categorised into closed (all forest classes) and open (fynbos, grasslands, savanna and succulent Karoo) habitat which was used as the character states for ancestral state reconstruction. Terminal taxa were coded accordingly and generalist taxa that occur in both closed and open habitat were coded to have a multistate. All genetic samples for *Philothamnus* and *Crotaphopeltis* were mapped onto the Terrestrial Ecoregions of the Afrotropical region (Olson et al., 2001) (Fig. 1), as well as biomes of South Africa (Mucina & Rutherford, 2006). Literature sources were additionally used to confirm classification of species into biome classes, in which they typically occur (Loveridge, 1958; Broadley, 1983; Hughes, 1985; Rasmussen, 1993; Rasmussen & Hughes, 1997; Rasmussen, Chirio & Ineich, 2000). Species from sister genera that are closely related to *Crotaphopeltis* and *Philothamnus* were coded using the same procedure, with locality data derived from Wallach, Williams & Boundy (2014). Species from sister genera that are closely related to *Crotaphopeltis* were coded as follows: *Boiga forsteni*, multistate, *Telescopus semiannulatus*, multistate; *Dipsadoboa unicolor*, closed habitat. Species from sister genera that are closely related to *Philothamnus* were coded as follows: *Thrasops jacksonii*, closed habitat; *Thelotornis kirtlandii*, closed habitat; *Dispholidus typus*, multistate and all three *Hapsidophrys* species, closed habitat.

## Results

**Figure 2:**
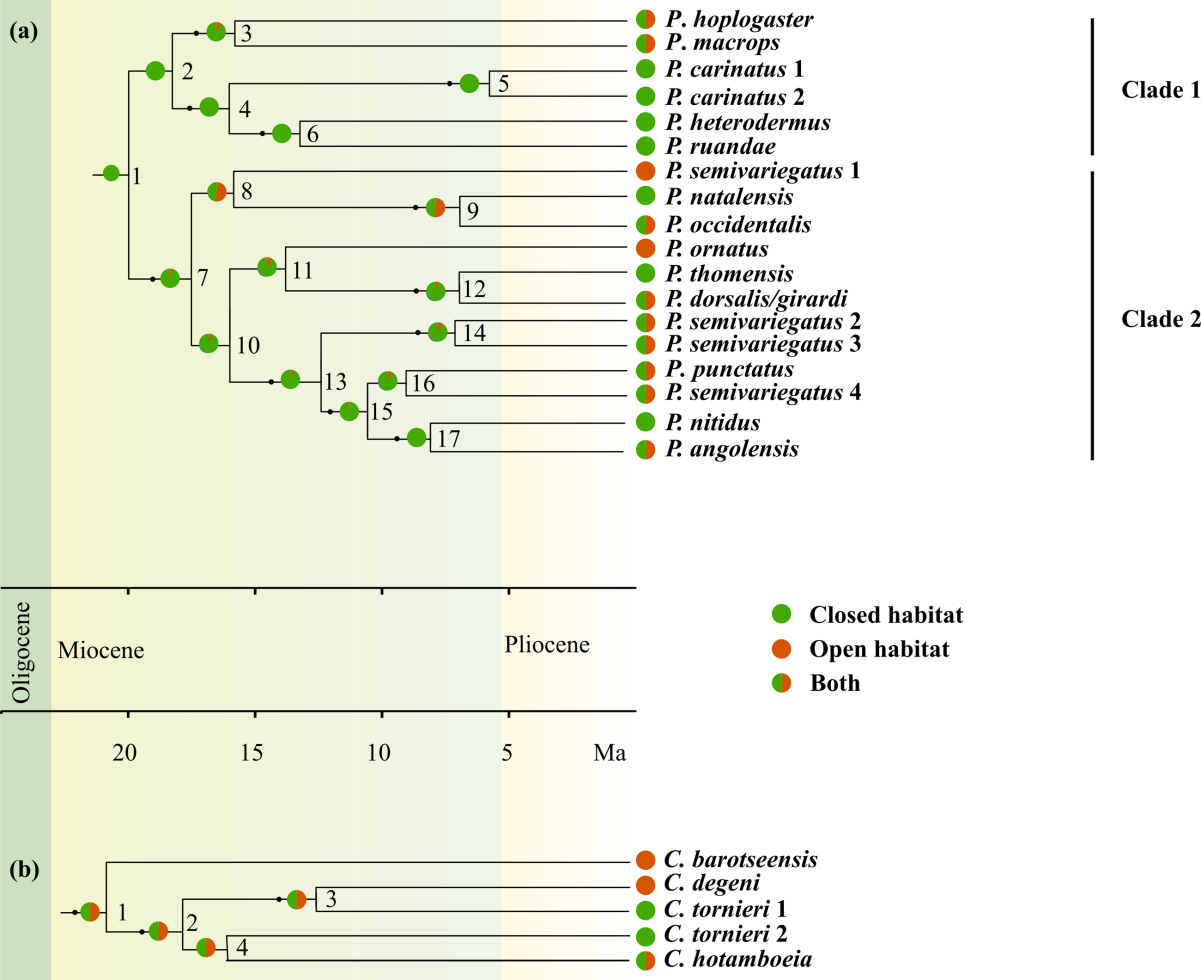
Chronograms for *Crotaphopeltis* and *Philothamnus,* with support values from the BEAST analysis of the unconstrained phylogeny (Fig. S1). Supported nodes (≥ 0.95 posterior probability) are indicated by black dots. Within *Philothamnus*, clade 1 denotes a group of species that comprise of predominantly forest species in the northern Afrotropical realm, and clade 2 a group of species that comprise open habitat specialists in southern Africa, as well as widespread habitat generalists in the rest of sub-Saharan Africa. Habitat reconstructions and terminal taxa are colour-coded according habitat classes; green (closed habitat), brown (open habitat) and green** +** brown (both open and closed habitats), with likelihood values for each state (Table 1) shown as a proportion in the pie charts at each node.

Phylogenetic results from the BEAST analysis were similar to findings in Engelbrecht et al. (2019); Engelbrecht et al. (2020), except for the unresolved placement of *P. semivariegatus* 1 in this study (Fig. 2A; node 8, Fig. S1). Similar to Engelbrecht et al. (2019), *P. semivariegatus* taxa were found to be paraphyletic. However, according to the latter study, *P*. *semivariegatus* 1 seems to be sister to a clade containing the three other *P*. *semivariegatus* taxa, *P. angolensis*, *P. nitidus* and *P. punctatus*. Divergence times and associated credibility intervals (Fig. S1) indicate that both *Crotaphopeltis* and *Philothamnus* diverged from their forest relatives, *D. unicolor* and *Hapsidophrys* spp. around the Late Oligocene.

Ancestral habitat reconstruction unequivocally indicates a closed habitat ancestral state for *Philothamnus* (0.99, Table 1; Fig. 2A, node 1). Lineage diversification estimation shows a major diversification event within this genus during Early Miocene (19.62 Mya; Fig. 2A, node 1). This initial divergence within *Philothamnus* resulted in two major clades, one that comprises species that have their distributions mainly in the northern regions of the Afrotropical realm, while the second clade comprises species that are distributed in the rest of sub-Saharan Africa (Clade 1 and 2, Fig. 2). Species from the northern Afrotropical realm (Clade 1) remained predominantly in closed habitat (*P. carinatus* 1, *P. carinatus* 2, *P. heterodermus*, *P. ruandae*) with the exception of *P. hoplogaster* and *P. macrops*. The two latter species diverged from their forest sister species during the Early/Mid-Miocene (17.9 Mya, Fig. 2A, node 2), occur in both closed and open habitat and are therefore considered the earliest diverging habitat generalist species in the genus. The majority of species in Clade 2 show several independent transitions into open habitat until the Pliocene. However, three species, *P. nitidus, P. thomensis*, *P. natalensis* within Clade 2 remained in closed habitat and diverged from their generalist sister taxa during the Late Miocene/Pliocene transition (7.75 Mya, Fig. 2A, node 17; 6.60 Mya, Fig. 2A, node 12; 6.58 Mya, Fig. 2A, node 9 respectively). Within the same clade, *P. ornatus* and *P. semivariegatus* 1 specialised into open habitat, since 17–13 Mya (Early/Mid-Miocene, Fig. 2A, node 11 and 8 respectively). *Philothamnus ornatus* appear to have a closed habitat ancestral state, while *P*. *semivariegatus* 1, seems to have specialised into open habitat from a mixed habitat state.

**Table 1:** Results from ancestral habitat reconstruction for *Crotaphopeltis* and *Philothamnus*. Likelihood values are indicated for closed and open habitat states at the respective nodes. Node numbers refer to Fig. 2.

Genus	Node number	Closed habitat		Open habitat	
*Philothamnus*	1	0.99		0.0074	
	2	0.99		0.0085	
	3	0.90		0.9500	
	4	0.99		0.0009	
	5	0.99		0.0003	
	6	0.99		0.0003	
	7	0.95		0.0490	
	8	0.48		0.524	
	9	0.48		0.5200	
	10	0.95		0.0460	
	11	0.90		0.0960	
	12	0.95		0.0510	
	13	0.96		0.0390	
	14	0.90		0.0960	
	15	0.97		0.0300	
	16	0.93		0.0690	
	17	0.50		0.0500	
*Crotaphopeltis*	1	0.49		0.5000	
	2	0.49		0.5000	
	3	0.50		0.4900	
	4	0.49		0.5000	

In contrast, the ancestral habitat type for *Crotaphopeltis* was equivocal (Table 1; Fig. 2B, node 1). *Crotaphopeltis barotseensis*, which is the earliest diverging species in the genus, specialised into open habitat during the Oligocene/Miocene transition (20.59 Mya, Fig. 2B, node 1). The second diversification event within *Crotaphopeltis* was between *C. degeni* + *C. tornieri* 1 and *C. tornieri* 2 + *C. hotamboeia* (17.59 Mya, Fig. 2B, node 2). *Crotaphopeltis degeni* diverged from *C. tornieri* 1 during the Mid-Miocene (12.34 Mya, Fig. 2B, node 3) and specialised into open habitat, while *C. tornieri* 1 moved into Afromontane forest habitat. The widespread *C. hotamboeia* showed no preference for either habitat type. Intraspecific diversification for *C. hotamboeia* commenced during the Late Miocene (6.45 Mya, Fig. S1). The sister relationship of *C*. *tornieri* 2 and *C*. *hotamboeia* is unsupported (similar to Engelbrecht et al., 2020), however this taxon appears to have diverged from its congeners during the Miocene period, as the inclusion within *C*. *degeni* + *C. tornieri* 1 + *C*. *hotamboeia* is supported (Fig. 2B, node 2). Similar to *C*. *tornieri* 1, *C*. *tornieri* 2 has specialised for Afromontane forest habitat.

## Discussion

*Crotaphopeltis* and *Philothamnus* show lineage diversification in the Mid-Miocene, most likely in relation to climatic oscillations and associated major biome shifts. Both genera diverged from their forest relatives during the Late Oligocene, a period that coincides with the initial contraction of the pan-African forest, due to global cooling and drier climate in equatorial Africa (Coetzee, 1993; Zachos et al., 2001; Couvreur et al., 2008; Couvreur et al., 2021; Kissling et al., 2012). The initial Early Miocene diversification within both genera post-dates the initial biome shift. Therefore, biome shift was probably not a driver of diversification because open habitats were already prevalent. *Philothamnus* appears to have inhabited forest, and the fragmentation of remaining forest patches in the Mid-Miocene probably isolated lineages that speciated in allopatry, with some generalist lineages utilising both habitat types. This has resulted in the radiation of the genus into at least 25 species. In contrast, *Crotaphopeltis* probably was not forest restricted, and possibly utilised both open and closed habitats, which precluded them from vicariance and speciation in allopatry. The result is far fewer lineages, mostly associated with open habitat, but with two independent moves into forest during the Mid-Miocene.

Initial divergence within *Philothamnus* gave rise to two major clades during the Early Miocene. Within one of these, a clade of four species (*P. carinatus* 1, *P. carinatus* 2, *P. heterodermus* and *P. ruandae*) seemed to have remained predominantly in closed habitat within the northern Afrotropical realm. Lineage diversification amongst these forest species seems to follow the east African and west/central biogeographic break observed for rainforest tree families as well as animal taxa (e.g., White, 1979; Wasser, 1993; Burgess & Clarke, 2000; Loader et al., 2007; Zimkus et al., 2016). Forest habitat is thought to have extended in a central belt running coast to coast during Early Miocene with fragmentation during Early/Mid-Miocene transition and again during Late Miocene, after the initiation of geological activity in the western east African Rift System (Axelrod & Raven, 1978; Wasser, 1993; Jacobs, Kingston & Jacobs, 1999; Jacobs, 2004; Sepulchre et al., 2006; Couvreur et al., 2008). Accordingly, the diversification dates amongst the four northern Afrotropical *Philothamnus* forest species correspond to the series of connection-isolation events between east African and Guinea-Congolian forests. Forest fragmentation during the Early/Mid-Miocene transition was concomitant with the advent of savanna in east Africa (Bobe, 2006; Couvreur et al., 2008), and was primarily driven by the aridification of the Congo basin (Senut, Pickford & Ségalen, 2009), the end of a warming trend (18–14 Mya) and onset of marked global cooling (Zachos et al., 2001). Therefore, the Early/Mid-Miocene savanna habitat in east Africa could have provided opportunities for species to radiate into open habitat, such as *Philothamnus hoplogaster* and *P. macrops*. These two species occur in both closed and open habitat, diverged from the northern Afrotropical forest species during the Early/Mid-Miocene and are therefore likely the oldest habitat generalists in the genus. *Philothamnus hoplogaster* and *P. macrops* have eastern Africa distributions, where the former is more widespread from east Africa to South Africa, and the latter is restricted to Tanzania (Wallach, Williams & Boundy, 2014). It is therefore suggested that *Philothamnus hoplogaster* and *P. macrops* radiated into open habitat with the concomitant advent of savanna and forest fragmentation in east Africa during the Early/Mid-Miocene transition (Bobe, 2006; Couvreur et al., 2008).

The second major clade within *Philothamnus* comprises a number of species that presumably diversified into open habitat. In fact, the *P. semivariegatus* 1 lineage could have become an open habitat specialist in southern Africa during the Early/Mid-Miocene transition, a time period when sclerophyll vegetation dominated the landscape of southern Africa (Axelrod & Raven, 1978). Most species in this *Philothamnus* clade are habitat generalists with relatively wide distributions. Since the Mid-Miocene, south-western Africa became more arid with upwelling of cold waters associated with the Benguela current (Siesser, 1978; Udeze & Oboh-Ikuenobe, 2005; Senut, Pickford & Ségalen, 2009). Thus, aridification of south-western Africa possibly contributed to a significantly more heterogeneous environment that could have facilitated a speciation pulse and radiation into novel habitats, especially into southern Africa. For example, *P. natalensis*, *P. nitidus* and *P. thomensis* remained in forest habitat, *P. ornatus* appears to have specialised into savanna habitat in southern Africa, while the majority of species became widespread habitat generalists.

*Crotaphopeltis* shows timing of lineage diversification that could be linked to the development of the savanna biome. Furthermore, lineage diversification within *Crotaphopeltis* corresponds with several divergence events within *Philothamnus*, although biogeographical patterns between the genera are different. For example, open habitat in most parts of southern Africa during the Early Miocene (Axelrod & Raven, 1978), possibly provided opportunities for open habitat (moist grasslands) specialisation of the oldest species within *Crotaphopeltis*, *C. barotseensis*. In contrast, there are two paraphyletic lineages of *C. tornieri*, in north-eastern and south-western Tanzania that are divergent at species level (Engelbrecht et al., 2020). Both occur in Afromontane forest at present, and probably represent secondary independent shifts from an open or mixed habitat ancestral state. During the same time period (Mid-Miocene), the specialisation of *C. degeni* for open habitat could have been induced by the presence of savanna habitats specifically in Ethiopia, Kenya and Uganda (Bobe, 2006). The fragmented occurrence of this species and morphological differentiation between populations of *C. degeni* (Rasmussen & Hughes, 1997), further supports specialisation into the patchy savanna environment of the Mid-Miocene East Africa.

The Miocene epoch signifies a period of increased diversity within both *Crotaphopeltis* and *Philothamnus*, which is similar to findings of other African reptile taxa (Wüster et al., 2007; Tolley, Chase & Forest, 2008; Tolley et al., 2011; Barlow et al., 2013; Ceccarelli et al., 2014; Menegon et al., 2014; Dowell et al., 2016; Medina et al., 2016; Barlow et al., 2019). Speciation patterns for the two genera furthermore fits the main diversification models documented for African savanna-forest ecosystems, such as the “Pleistocene lowland forest refugia” (geographic model) and “vanishing refugia” (ecological model) mechanisms (Couvreur et al., 2021). *Philothamnus* shows notably more cladogenic events relative to *Crotaphopeltis*, irrespective of similar emergence times for the two genera. Given that *Philothamnus* has an ancestral preference for forest habitat, the fragmentation of this biome since the Oligocene must have had a greater evolutionary impact on the distribution of species in this genus than it did on *Crotaphopeltis* that have no preference for either closed or open habitat.

The inclusion of missing taxa in biogeographical analyses (eight *Philothamnus* and two *Crotaphopeltis* species) may support alternative hypotheses with regards to the radiation patterns for each of the snake genera. Especially, for species-specific cases and given the sensitivity of ancestral state reconstruction methods to the distribution of character states within a clade (Holland et al., 2020). However, the overarching biogeographical pattern for each genus may remain, given the sub-Saharan African landscape dynamics and the distinct life-history traits of *Crotaphopeltis* and *Philothamnus*. Furthermore, the evolution of habitat generalist species in *Philothamnus* since the Early Miocene is in agreement with the hypothesis that the origins of generalist species are triggered by a physically and temporally variable environment (Futuyma & Moreno, 1988; Remold, 2012). Radiation of *Philothamnus* and *Crotaphopeltis* species into novel habitats are possibly facilitated by ecological opportunity (Losos & Mahler, 2010) associated with the development of major biomes since the Oligocene.

##  Supplemental Information

10.7717/peerj.11728/supp-1 Supplemental Information 1 Approximate geographical range and habitat type association for Crotaphopeltis and Philothamnus species included in ancestral habitat reconstruction analysis Capital letters, “N”, “E”, “S” and “W”, “NE”, “SE”, “SW” and “NW” refer to cardinal and intercardinal directions. Habitat type was classified according to theTerrestrial Ecoregions and further categorised as follows: all forest classes = closed habitat; fynbos, succulent Karoo, grasslands and savanna = open habitat. Sources: (Broadley, 1983; Olson et al., 2001; Wallach, Williams & Boundy, 2014; Engelbrecht et al., 2019; Engelbrecht et al., 2020).

10.7717/peerj.11728/supp-2 Supplemental Information 2 Sample information for lineage diversification estimation analyses of *Crotaphopeltis* and *Philothamnus* species Additional species were included as outgroup taxa and to provide a temporal framework. GenBank accession numbers are provided, where NA denotes missing information.

10.7717/peerj.11728/supp-3 Supplemental Information 3 Time calibrated Bayesian inference tree for *Crotaphopeltis* and *Philothamnus* species The tree was inferred from the concatenated dataset of cyt–b, ND4 and c–mos gene fragments, based on three calibration points (see Material and Methods). Mean age estimates are provided near the nodes with bars indicating the 95% highest posterior densities (HPD). Black circles denote posterior probability values ≥ 0.95. Sample codes correlate to specimens in Table S2.

10.7717/peerj.11728/supp-4 Supplemental Information 4 BEAST input file for lineage diversification analysis

10.7717/peerj.11728/supp-5 Supplemental Information 5 BEAST lineage diversification meanHeights tree file

10.7717/peerj.11728/supp-6 Supplemental Information 6 Ancestral habitat reconstruction (Mesquite) results, analysed on the BEAST subtree for Crotaphopeltis

10.7717/peerj.11728/supp-7 Supplemental Information 7 Ancestral habitat reconstruction (Mesquite) results, analysed on the BEAST subtree for Philothamnus

10.7717/peerj.11728/supp-8 Supplemental Information 8 BEAST text file

## Additional Information and Declarations

### Data Availability

The following information was supplied regarding data availability: The raw data are available in the Supplemental Files.
