# Supplementary material for: Snakes on an African plain: the radiation of Crotaphopeltis and Philothamnus into open habitat (Serpentes: Colubridae)
Source: PeerJ. 2021 Aug 6;9:e11728. doi: 10.7717/peerj.11728 (PMC8351568; doi:10.7717/peerj.11728)
Supplement: Supplemental Information 1 — Capital letters, “N”, “E”, “S” and “W”, “NE”, “SE”, “SW” and “NW” refer to cardinal and intercardinal directions. Habitat type was classified according to theTerrestrial Ecoregions and further categorised as follows: all forest classes = closed habitat; fynbos, succulent Karoo, grasslands and savanna = open habitat. Sources: (Broadley, 1983; Olson et al., 2001; Wallach, Williams & Boundy, 2014; Engelbrecht et al., 2019; Engelbrecht et al., 2020). [file peerj-09-11728-s001.docx]

**Supplementary Table 1.** Approximate geographical range and habitat type association for Crotaphopeltis and Philothamnus species included in ancestral habitat reconstruction analysis. Capital letters, “N”, “E”, “S” and “W”, “NE”, “SE”, “SW” and “NW” refer to cardinal and intercardinal directions. Habitat type was classified according to theTerrestrial Ecoregions and further categorised as follows: all forest classes = closed habitat; fynbos, succulent Karoo, grasslands and savanna = open habitat. Sources: Broadley, 1983; Olson et al., 2001, Wallach, Williams & Boundy, 2014; Engelbrecht et al. 2019; 2020.

| Species | Approximate geographical range | Habitat association/specialisation | |
| --- | --- | --- | --- |
|  |  |  |  |
| *Crotaphopeltis* (n = 16) |  |  |  |
|  |  |  |  |
| *C*. *barotseensis* (n = 1) | Southern Africa  (W Zambia, NE Namibia and NW Botswana) | Open |  |
|  |  |  |  |
| *C. degeni* (n = 1) | Central Africa  (N Cameroon, N Central African Republic, S Sudan, E South Sudan, W Ethiopia, S Uganda, SW Kenya and SW Tanzania) | Open |  |
|  |  |  |  |
| *C. hotamboeia* (n = 11) | Central, East, Southern and West Africa  (SW Mauritania, Senegal, Gambia, Guinea-Bissau, Guinea, Sierra Leone, Liberia, Ivory Coast, SW Mali, Burkina Faso, Togo, Benin, Niger, Nigeria, S Chad, Cameroon, Central African Republic, Gabon, Congo, Democratic Republic of Congo, NE Angola, SE Sudan, Eritrea, Ethiopia. S Somalia, Kenya, Angola, Uganda, Rwanda, Burundi, Tanzania, Zambia, Malawi, Mozambique, Zimbabwe, E Botswana, N Namibia, Swaziland, Lesotho and SE South Africa) | Generalist |  |
|  |  |  |  |
| *C. tornieri* 1 (n = 2) | East Africa  (cen. and NE Tanzania (Engelbrecht et al. 2020)) | Closed |  |
| *C. tornieri* 2 (n = 1) | East Africa  (SW Tanzania (Engelbrecht et al. 2020)) | Closed |  |

| Supplementary Table 1. (continue) | | |  |
| --- | --- | --- | --- |
| Species | **Approximate geographical range** | **Habitat association/specialisation** | |
|  |  |  |  |
| *Philothamnus* (n = 29) |  |  |  |
|  |  |  |  |
| *P*. *angolensis* (n = 9) | Central, East Africa and Southern Africa  (Cameroon, W Central African Republic, Democratic Republic of Congo, Sudan, E South Sudan, E South Sudan, Uganda, Rwanda, Burundi, Tanzania, N NW and W Zambia, Malawi, Mosambique, Zimbababwe, Angola, cen. Namibia, N Botswana and NE South Africa) | Generalist |  |
|  |  |  |  |
| *P*. *carinatus* 1 (n = 1) | Central Africa  (E Democratic Republic of Congo) | Closed |  |
|  |  |  |  |
| *P*. *carinatus* 2 (n = 1) | West Africa  (Gabon and Congo-Brazzaville) | Closed |  |
|  |  |  |  |
| *P*. *dorsalis/girardi* (n = 2) | Predominantly Central Africa/ W Africa  (S Cameroon, W Gabon, S Congo, Democratic Republic of Congo and N Angola/ Equatorial Guinea) | Generalist |  |
|  |  |  |  |
| *P*. *heterodermus* (n = 1) | Predominantly West and Central Africa  (Guinea-Bissau, Guinea, S Sierra Leone, S Ivory Coast, S Ghana, S Togo, S Benin, S Nigeria, Cameroon, S Chad, Central African Republic, Gabon, Congo, Democratic Republic of Congo, SW Uganda, N Rwanda, Burundi, Tanzania and W Angola) | Closed |  |
|  |  |  |  |

| Supplementary Table 1. (cont.) | | |  |
| --- | --- | --- | --- |
| Species | **Approximate geographical range** | **Habitat association/specialisation** | |
| *P*. *hoplogaster* (n = 1) | East and Southern Africa  (SE South Sudan, S Democratic Republic of Congo, S Uganda, Tanzania, Angola, Zambia, Malawi, Mozambique, Zimbabwe, NE Botswana,  cen. Namibia, Swaziland and South Africa) | Generalist |  |

| *P*. *macrops* (n = 1) | East Africa  (E Tanzania) | Generalist |
| --- | --- | --- |
|  |  |  |
| *P*. *natalensis* (n = 1) | Southeastern Africa  (S Mozambique, SE Zimbabwe, E Swaziland and NE South Africa) | Closed |
|  |  |  |
| *P*. *nitidus* (n = 2) | Predominantly West, Central and East Africa  (SE Guinea, S Sierra Leone, S Ivory Coast, S Ghana, S Togo, S Benin, SW Nigeria, S Cameroon, SW Central African Republic, Equatorial Guinea, Gabon, Congo, Democratic Republic of Congo, NE Angola, S Uganda, Rwanda, Burundi and SW Tanzania) | Closed |
|  |  |  |
| *P*. *occidentalis* (n = 2) | Southern Africa  (Inland NE South Africa, S South Africa and NW Swaziland) | Generalist |
| *P*. *ornatus* (n = 1) | Predominantly Central Africa and Southern Africa  (S Cameroon, NE Democratic Republic of Congo, SW Tanzania, W Angola, NW Zambia, NE Namibia, N Botswana and NE Zimbabwe) | Open |

| Supplementary Table 1. (cont.) | | |  |
| --- | --- | --- | --- |
| Species | **Approximate geographical range** | **Habitat association/specialisation** | |
| *P*. *punctatus* (n = 1) | Predominantly East Africa  (E Ethiopia, Somalia, E Kenya, E Tanzania, NE Malawi and N Mozambique) | Generalist |  |
|  |  |  |  |
| *P*. *ruandae* (n = 1) | East Africa  (E Democratic Republic of Congo, W Rwanda and NW Burundi) | Closed |  |
|  |  |  |  |
| *P*. *semivariegatus* 1 (n = 1) | Southern Africa  (N-cen. South Africa (Engelbrecht et al. 2019)) | Open |  |
|  |  |  |  |
| *P*. *semivariegatus* 2 (n = 1) | Southern Africa  (NE South Africa (Engelbrecht et al. 2019)) | Generalist |  |
|  |  |  |  |
| *P*. *semivariegatus* 3 (n = 1) | East and Southern Africa  (SE Africa (Engelbrecht et al. 2019)) | Generalist |  |
|  |  |  |  |
| *P*. *semivariegatus* 4 (n = 1) | Central Africa (as per Engelbrecht et al. 2019) | Generalist |  |
|  |  |  |  |
| *P*. *thomensis* (n = 1) | São Tomé and Príncipe islands | Closed |  |
