# Supplementary material for: Snakes on an African plain: the radiation of Crotaphopeltis and Philothamnus into open habitat (Serpentes: Colubridae)
Source: PeerJ. 2021 Aug 6;9:e11728. doi: 10.7717/peerj.11728 (PMC8351568; doi:10.7717/peerj.11728)
Supplement: Supplemental Information 2 — Additional species were included as outgroup taxa and to provide a temporal framework. GenBank accession numbers are provided, where NA denotes missing information. [file peerj-09-11728-s002.docx]

**Supplementary Table 2.** Sample information for lineage diversification estimation analyses of *Crotaphopeltis* and *Philothamnus* species. Additional species were included as outgroup taxa and to provide a temporal framework. GenBank accession numbers are provided, where NA denotes missing information.

| Family (Subfamily) | *Genus species* | *c–mos* | *cyt–b* | ND4 |
| --- | --- | --- | --- | --- |
| Colubridae (Colubrinae) | *Bamanophis dorri* | NA | NA | AY188001 |
| Colubridae (Colubrinae) | *Boiga dendrophila* | AF471128 | AF471089 | U49303 |
| Colubridae (Colubrinae) | *Boiga forsteni* | KC347388 | KC347468 | KC347506 |
| Colubridae (Calamariinae) | *Calamaria pavimentata* | KX694804 | KX694890 | NA |
| Colubridae (Colubrinae) | *Coronella girondica* | AF471113 | AF471088 | AY487066 |
| Colubridae (Colubrinae) | *Crotaphopeltis barotseensis* | MN102586 | MN102494 | MN102563 |
| Colubridae (Colubrinae) | *Crotaphopeltis degeni* | NA | MN102495 | MN102562 |
| Colubridae (Colubrinae) | *Crotaphopeltis hotamboeia* | MN102588 | MN102448 | MN102524 |
| Colubridae (Colubrinae) | *Crotaphopeltis hotamboeia* | MN102571 | MN102460 | MN102501 |
| Colubridae (Colubrinae) | *Crotaphopeltis hotamboeia* | MN102604 | MN102454 | MN102558 |
| Colubridae (Colubrinae) | *Crotaphopeltis hotamboeia* | MN102593 | NA | MN102552 |
| Colubridae (Colubrinae) | *Crotaphopeltis hotamboeia* | MN102600 | MN102473 | MN102531 |
| Colubridae (Colubrinae) | *Crotaphopeltis hotamboeia* | MN102580 | MN102443 | MN102518 |
| Colubridae (Colubrinae) | *Crotaphopeltis hotamboeia* | MN102592 | MN102491 | MN102554 |
| Colubridae (Colubrinae) | *Crotaphopeltis hotamboeia* | MN102585 | MN102484 | MN102542 |
| Colubridae (Colubrinae) | *Crotaphopeltis hotamboeia* | MN102582 | MN102479 | MN102540 |
| Colubridae (Colubrinae) | *Crotaphopeltis hotamboeia* | MN102579 | MN102485 | MN102557 |
| Colubridae (Colubrinae) | *Crotaphopeltis hotamboeia* | MN102566 | MN102478 | MN102539 |
| Colubridae (Colubrinae) | *Crotaphopeltis tornieri* 1 | NA | AF428034 | NA |
| Colubridae (Colubrinae) | *Crotaphopeltis tornieri* 1 | NA | AF428031 | NA |
| Colubridae (Colubrinae) | *Crotaphopeltis tornieri* 2 | NA | MN102493 | MN102564 |
| Colubridae (Colubrinae) | *Dipsadoboa unicolor* | MN102606 | MN102492 | MN102498 |
| Colubridae (Colubrinae) | *Dispholidus typus* | MH756226 | NA | MH756532 |
| Colubridae (Colubrinae) | *Dispholidus* sp. | MH756205 | MH756446 | MH756509 |
| Colubridae (Colubrinae) | *Dolichophis caspius* | AY376797 | AY039173 | AY487039 |
| Colubridae (Colubrinae) | *Dolichophis jugularis* | AY376798 | AY376740 | AY487046 |
| Colubridae (Colubrinae) | *Eirenis eiselti* | NA | NA | AY487069 |
| Colubridae (Colubrinae) | *Eirenis levantinus* | AY376823 | AY376765 | AY487071 |
| Colubridae (Colubrinae) | *Eirenis modestus* | AY486957 | AY486933 | AY487072 |

| **SupplementaryTable 1.** (*continued*) |
| --- |

| Family (Subfamily) | *Genus species* | *c–mos* | *cyt–b* | ND4 |
| --- | --- | --- | --- | --- |
| Colubridae (Colubrinae) | *Eirenis punctatolineatus* | AY376813 | AY376755 | AY487073 |
| Colubridae (Colubrinae) | *Elaphe quatuorlineata* | AY486955 | AY486931 | AY487067 |
| Colubridae (Grayiinae) | *Grayia tholloni* | DQ486175 | DQ486351 | DQ486326 |
| Colubridae (Colubrinae) | *Hapsidophrys lineatus* | MH756203 | MH756445 | MH756507 |
| Colubridae (Colubrinae) | *Hapsidophrys principis* | NA | FJ913492 | NA |
| Colubridae (Colubrinae) | *Hapsidophrys smaragdinus* | MH756202 | MH756444 | MH756506 |
| Colubridae (Colubrinae) | *Hemerophis socotrae* | AY188003 | AY188042 | NA |
| Colubridae (Colubrinae) | *Hemorrhois algirus* | AY486935 | AY486911 | AY487037 |
| Colubridae (Colubrinae) | *Hemorrhois hippocrepis* | AY486940 | AY486916 | AY487045 |
| Colubridae (Colubrinae) | *Hemorrhois nummifer* | AY376800 | AY376742 | AY487049 |
| Colubridae (Colubrinae) | *Hemorrhois ravergieri* | AY486944 | AY486920 | AY487050 |
| Colubridae (Colubrinae) | *Hierophis gemonensis* | AY376799 | AY039183 | LN552101 |
| Colubridae (Colubrinae) | *Hierophis viridiflavus* | AY486949 | AY486925 | FJ430660 |
| Colubridae (Colubrinae) | *Pantherophis bairdi* | DQ902061 | GU073395 | NA |
| Colubridae (Colubrinae) | *Pantherophis obsoletus* | FJ627805 | DQ538343 | KM655218 |
| Colubridae (Colubrinae) | *Pantherophis spiloides* | NA | NA | KM655241 |
| Colubridae (Colubrinae) | *Philothamnus angolensis* | MH756206 | NA | MH756510 |
| Colubridae (Colubrinae) | *Philothamnus angolensis* | MH756208 | MH756448 | MH756512 |
| Colubridae (Colubrinae) | *Philothamnus angolensis* | MH756216 | NA | MH756524 |
| Colubridae (Colubrinae) | *Philothamnus angolensis* | NA | NA | MH756593 |
| Colubridae (Colubrinae) | *Philothamnus angolensis* | MH756246 | MH756470 | MH756551 |
| Colubridae (Colubrinae) | *Philothamnus angolensis* | MH756248 | MH756472 | MH756553 |
| Colubridae (Colubrinae) | *Philothamnus angolensis* | MH756249 | NA | MH756555 |
| Colubridae (Colubrinae) | *Philothamnus angolensis* | NA | NA | MH756597 |
| Colubridae (Colubrinae) | *Philothamnus angolensis* | NA | MH756495 | MH756589 |
| Colubridae (Colubrinae) | *Philothamnus carinauts* 1 | MH756199 | NA | MH756503 |
| Colubridae (Colubrinae) | *Philothamnus carinatus* 2 | MH756241 | MH756467 | MH756546 |
| Colubridae (Colubrinae) | *Philothamnus dorsalis/girardi* | MH756234 | MH756462 | MH756540 |
| Colubridae (Colubrinae) | *Philothamnus dorsalis/girardi* | NA | FJ913495 | NA |
| Colubridae (Colubrinae) | *Philothamnus heterodermus* | MH756235 | MH756463 | MH756541 |
| Colubridae (Colubrinae) | *Philothamnus hoplogaster* | MH756247 | MH756471 | MH756552 |
| Colubridae (Colubrinae) | *Philothamnus macrops* | MH756280 | MH756493 | MH756587 |

| **Supplementary Table 1.** (*continued*) |
| --- |

| Family (Subfamily) | *Genus species* | *c–mos* | *cyt–b* | ND4 |
| --- | --- | --- | --- | --- |
| Colubridae (Colubrinae) | *Philothamnus natalensis* | MH756258 | MH756479 | MH756565 |
| Colubridae (Colubrinae) | *Philothamnus nitidus* | MH756222 | NA | MH756529 |
| Colubridae (Colubrinae) | *Philothamnus nitidus* | MH756238 | NA | MH756542 |
| Colubridae (Colubrinae) | *Philothamnus occidentalis* | MH756267 | MH756483 | MH756576 |
| Colubridae (Colubrinae) | *Philothamnus occidentalis* | MH756271 | MH756487 | MH756579 |
| Colubridae (Colubrinae) | *Philothamnus ornatus* | MH756264 | MH756481 | MH756570 |
| Colubridae (Colubrinae) | *Philothamnus punctatus* | MH756196 | NA | MH756500 |
| Colubridae (Colubrinae) | *Philothamnus ruandae* | MH756250 | MH756474 | MH756556 |
| Colubridae (Colubrinae) | *Philothamnus semivariegatus* 1 | MH756224 | MH756456 | MH756531 |
| Colubridae (Colubrinae) | *Philothamnus semivariegatus* 2 | MH756229 | MH756460 | MH756535 |
| Colubridae (Colubrinae) | *Philothamnus semivariegatus* 3 | MH756254 | MH756478 | MH756561 |
| Colubridae (Colubrinae) | *Philothamnus semivariegatus* 4 | MH756207 | MH756447 | MH756511 |
| Colubridae (Colubrinae) | *Philothamnus thomensis* | FJ913490 | NA | NA |
| Colubridae (Colubrinae) | *Pituophis catenifer* | FJ627790 | NA | JF308311 |
| Colubridae (Colubrinae) | *Pituophis catenifer vertebralis* | FJ627789 | FJ627819 | AF141126 |
| Colubridae (Colubrinae) | *Pituophis deppei* | KX694814 | FJ627818 | AF138766 |
| Colubridae (Colubrinae) | *Pituophis lineaticollis* | FJ627804 | NA | AF138768 |
| Colubridae (Colubrinae) | *Pituophis melanoleucus* | FJ627797 | AF337100 | EU272832 |
| Colubridae (Colubrinae) | *Pituophis ruthveni* | DQ902092 | NA | KJ938643 |
| Colubridae (Colubrinae) | *Platyceps collaris* | AY486946 | AY486922 | AY487053 |
| Colubridae (Colubrinae) | *Platyceps karelini* | AY486942 | AY486918 | AY487047 |
| Colubridae (Colubrinae) | *Platyceps najadum* | AY486936 | AY486912 | AY487048 |
| Colubridae (Colubrinae) | *Platyceps rhodorachis* | AY486945 | AY486921 | AY487051 |
| Colubridae (Colubrinae) | *Platyceps rogersi* | AY188002 | AY188041 | AY487052 |
| Colubridae (Colubrinae) | *Ptyas mucosa* | AF471151 | AF471054 | AY487063 |
| Colubridae (Colubrinae) | *Scaphiophis albopunctatus* | DQ486169 | DQ486345 | DQ486321 |
| Colubridae (Colubrinae) | *Sibynophis subpunctatus* | KC347411 | KC347471 | KC347516 |
| Colubridae (Colubrinae) | *Spalerosophis diadema* | AF471155 | AF471049 | AY487059 |
| Colubridae (Colubrinae) | *Telescopus semiannulatus* | MN102605 | MN102496 | MN102497 |
| Colubridae (Colubrinae) | *Thelotornis kirtlandii* | MH756219 | NA | MH756527 |
| Colubridae (Colubrinae) | *Thrasops jacksonii* | MH756221 | NA | MH756528 |
| Total | 92 | 79 | 72 | 84 |
