# Supplementary figures and images for: Snakes on an African plain: the radiation of Crotaphopeltis and Philothamnus into open habitat (Serpentes: Colubridae)
Source: PeerJ. 2021 Aug 6;9:e11728. doi: 10.7717/peerj.11728 (PMC8351568; doi:10.7717/peerj.11728)

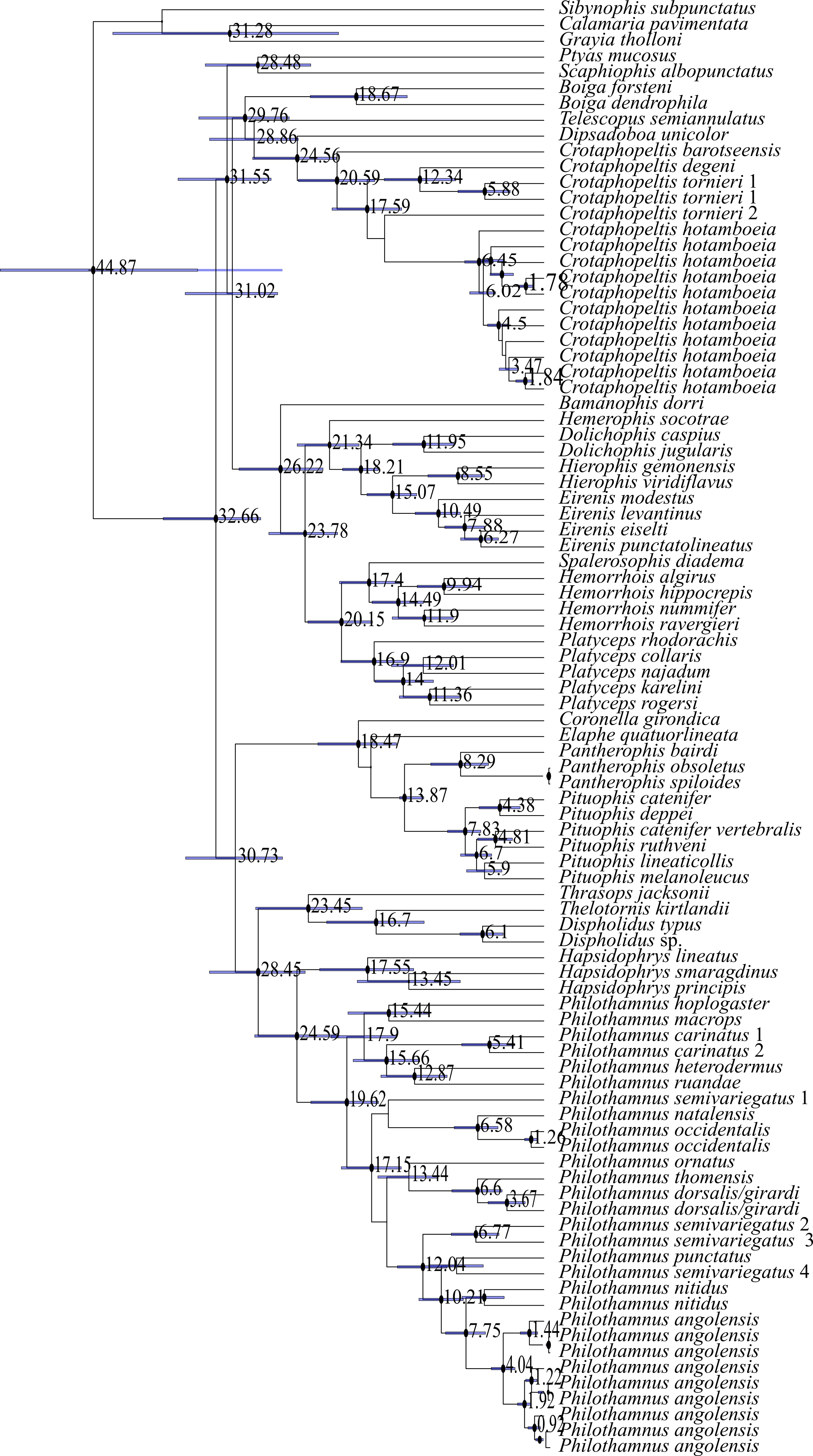

Supplement: Supplemental Information 3 — The tree was inferred from the concatenated dataset of cyt–b, ND4 and c–mos gene fragments, based on three calibration points (see Material and Methods). Mean age estimates are provided near the nodes with bars indicating the 95% highest posterior densities (HPD). Black circles denote posterior probability values ≥ 0.95. Sample codes correlate to specimens in Table S2. [file peerj-09-11728-s003.png]
